# Granular Immunofluorescence deposition of IgG in anti-GBM antibody disease

**DOI:** 10.11604/pamj.2014.17.296.3373

**Published:** 2014-04-18

**Authors:** Syed Amer, Mohammed Muqeetadnan

**Affiliations:** 1 Department of Internal Medicine, Brookdale University Hospital and Medical Center, Brooklyn, NY -11212, USA; 2 Department of Internal Medicine, University of Oklahoma Health Sciences Center, Oklahoma City, OK, 73104, USA

**Keywords:** IgG, anti-GBM antibody disease, Immunofluorescence

## Image in medicine

Our patient was a 75 year old male, who presented with worsening dysphagia for solids and liquids and increasing malaise. His past medical history was significant for Chronic Obstructive Pulmonary Disease (COPD), Coronary Artery Disease (CAD) and Peripheral Vascular Disease (PVD). He was then admitted and was found to have a creatinine of 3.5, with an unknown baseline. He was worked up for acute kidney injury and dysphagia. Esophagogastroduodenoscopy (EGD) was done and he was found to have achlasia. Urinalysis was done and it showed showed 3+ proteinuria. Subsequently, his creatinine peaked from 3.5 to 9.9. Serology for Anti-neutrophil cytoplasmic antibody (ANCA) was negative, but Anti-glomerular basement membrane antibody (anti-GBM Ab) was positive, with a titer of 184. Renal biopsy done,which was consistent with granular GBM than the usual linear pattern. He was subsequently started on stress dose of steroids. He did not show any response to steroids and so he was also started on cyclophosphamide and plasmapheresis was also done. Unfortunately, he did not respond to any of these therapies. He started deteriorating and soon developed shortness of breath, hemoptysis and diffuse alveolar hemorrhage. He was then admitted in the intensive care unit (ICU) and the decision was made to start dialysis, cyclophosphamide was stopped and the steroids were slowly tapered. The patient gradually improved with dialysis. This case demonstrates a very rare pattern of immunofluorescence in a patient with anti-GBM antibody disease. Normally, the immunofluorescence microscopy in these patients shows characteristic linear deposition of IgG. But our patient had a granular immunofluorescence pattern, a very rare finding in a patient with Goodpasture's disease. To our knowledge this is the first reported case with such an immunofluorescence pattern in a patient with Goodpasture's disease in the entire medical literature.

**Figure 1:**
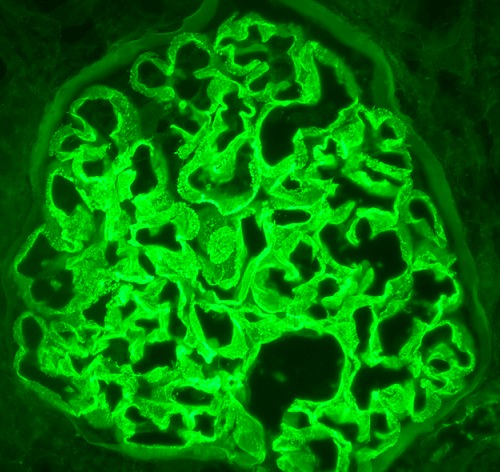
Granular pattern of IgG deposition seen. Usually a linear deposition of the antibodies is seen

